# Improving the Cycling Stability of Fe_3_O_4_/NiO Anode for Lithium Ion Battery by Constructing Novel Bimodal Nanoporous Urchin Network

**DOI:** 10.3390/nano10091890

**Published:** 2020-09-21

**Authors:** Xiaomin Zhang, Xiaoli Liu, Jun Zhou, Chunling Qin, Zhifeng Wang

**Affiliations:** 1 School of Materials Science and Engineering, Hebei University of Technology, Tianjin 300401, China; xmzhang1223@163.com (X.Z.); clqin@hebut.edu.cn (C.Q.); 2 Key Laboratory for New Type of Functional Materials in Hebei Province, Hebei University of Technology, Tianjin 300401, China; 3 School of Materials Science and Engineering, Hebei University of Science & Technology, Shijiazhuang 050018, China; iven308@126.com

**Keywords:** lithium ion battery, anode, dealloying, nanoporous

## Abstract

The development of facile preparation methods and novel three-dimensional structured anodes to improve cycling stability of lithium ion batteries (LIBs) is urgently needed. Herein, a dual-network ferroferric oxide/nickel oxide (Fe_3_O_4_/NiO) anode was synthesized through a facile dealloying technology, which is suitable for commercial mass manufacturing. The dual-network with high specific surface area contains a nanoplate array network and a bimodal nanoporous urchin network. It exhibits excellent electrochemical performance as an anode material for LIB, delivering a reversible capacity of 721 mAh g^−1^ at 100 mA g^−1^ after 100 cycles. The good lithium storage performance is related to the ample porous structure, which can relieve stress and mitigate the volume change in the charge/discharge process, the interconnected porous network that enhances ionic mobility and permeability, and synergistic effects of two kinds of active materials. The paper provides a new idea for the design and preparation of anode materials with a novel porous structure by a dealloying method and may promote the development of the dealloying field.

## 1. Introduction

Li-ion batteries (LIBs) have been widely used in various electronic equipment due to their high energy density and long service life [1,2,3,4]. The theoretical capacity of the currently used graphite anode is only 372 mAh g^−1^, which can no longer meet the market demand [5]. Therefore, the exploitation of new replaceable anode materials has become the focus of current studies [6,7]. Among various anode materials, Fe_3_O_4_ receives widespread attention because of its high theoretical capacity (926 mAh g^−1^) [8], easy availability, and environmental benignity [9,10]. At the same time, some disadvantages, such as poor conductivity, serious volume expansion, and poor cycle stability, also limit its application in high-performance LIBs [11,12]. Many strategies have been used to alleviate and restrain the above shortcomings. Multishell graphitic carbon-coated Fe_3_O_4_ hollow nanopowders [9] were synthesized by spray drying and oxidation process. Three-dimensional (3D) porous graphene nanoscroll/nanosheet supported Fe_1−x_S/Fe_3_O_4_ hetero-nanoparticles [11] were fabricated by cold quenching, freeze drying and the subsequent roll-in treatment. N-doped carbon coated Fe_3_O_4_ hollow spheres [13] were prepared by hydrothermal and magnetic self-assembly method. These materials showed excellent electrochemical performance as LIB anodes. However, most of the reported preparation methods are complicated, which are not conducive to commercial large-scale production.

Among all the possible attempts, dealloying has been verified to be a relatively facile method to fabricate nanoporous anode materials with excellent energy storage performance [14,15,16] by reasonably designing the precursor’s components and controlling corrosion conditions. With the assistance of a melting furnace and a holding furnace, precursor ribbons weighing up to 30 kg can be prepared by means of continuous melt-spinning method [17]. Then, these ribbons can be produced into active materials through chemical dealloying in batches. Three popular three-dimensional (3D) structures of dealloyed products, including nanoporous metals (Ge, Sn) with bi-continuous network-like structures [14,18], transition metal oxides nanosheet arrays [17,19] and dual-network nanoporous materials [3,20], have presented good Li storage properties. It was found that when the two sets of networks consisting of bimodal nanoporous network and nanosheet array network intertwine with each other to form a dual-network nanoporous structure, two networks in different size ranges can further fill the space of each other, so that the specific surface area of the material can be significantly increased and the transport distance of lithium ions can be reduced, inducing an improvement in the electrochemical performance [3]. In view of this, can bi-continuous bimodal nanoporous structures continue to evolve to other 3D structures in the process of dealloying? In addition, can we design and fabricate 3D dual-networks that are more conducive by dealloying method to facilitate the improvement in cyclic stability? Relevant efforts in this area are urgently needed. In the current study, we developed a facile dealloying method to successfully fabricate a novel dual-network Fe_3_O_4_/NiO anode, composed of a nanoplate network array and a bimodal nanoporous urchin network. The as-prepared composite shows improved cycling stability for LIBs after introducing the bimodal nanoporous urchin network, which offers an idea for designing advanced anodes with novel 3D structured frameworks.

## 2. Materials and Methods

**Figure 1:**
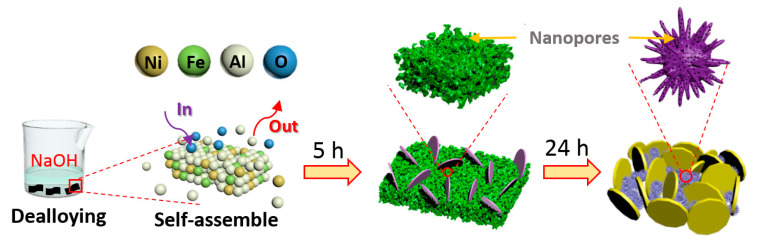
Schematic illustration showing the synthesis process of the dual-network nanoporous Fe_3_O_4_/NiO composites.

The Ni_5_Fe_5_Al_90_ (at.%) ingot was fabricated by arc-melting high purity Ni, Fe and Al (99.99 wt.%) ingots. The Ni_5_Fe_5_Al_90_ ribbons were obtained by a previously reported melt-spinning method [21,22,23]. The dealloying [24] was carried out by removing Al from precursor ribbons accompanied by self-assembly and spontaneous oxidation [25]. Typically, 3 g ribbons were dealloyed in 500 mL 2 M NaOH solution at 25 °C for 24 h. During the dealloying, the most active Al elements were removed, a part of the remaining Fe formed a Fe_3_O_4_ nanoplate network, while another part of Fe mixed with Ni created a nanoporous Fe_3_O_4_/NiO network. After washing several times with deionized water (DI-water) and drying in a vacuum oven at 60 °C for 12 h, the Fe_3_O_4_/NiO composites were obtained in pure form. The dealloying process is schematically presented in Figure 1.

The phase composition was analyzed by X-ray diffraction (XRD, Bruker D8, Karlsruhe, Germany), using Cu Kα radiation. The morphologies of the products were observed by scanning electron microscopy (SEM, Hitachi S-4800, Tokyo, Japan) and transmission electron microscopy (TEM, JEM 2100, Tokyo, Japan). The specific surface areas and pore size distribution of the products were obtained by the Brunauer–Emmett–Teller (BET) method and the Barrett–Joyner–Halenda (BJH) method, respectively. X-ray photoelectron spectroscopy (XPS, V-Sorb 2800P, Beijing, China) was used to reveal the valence states of superficial elements.

A mixture of active materials (70%), Ketjen black (20%) and carboxymethyl cellulose (10%) binder was dispersed in DI-water and grinded for 40 min to form a slurry. The slurry was bladed on copper foils and dried in a vacuum drying oven. Then, the working electrode was obtained by punching out 10 mm diameter disks and was assembled in coin-type cells (CR2032 model, Xinghua Benote Battery Material Co., Ltd., Xinghua, China) in an argon-filled glove box. Half cells were fabricated with Li foil as counter electrode and Celgard 2400 was used as the separator. Then, 1 M LiPF_6_ in ethylene carbonate/dimethyl carbonate (1:1, *v*/*v*) was used as the electrolyte (Shanghai Xiaoyuan Energy Technology Co., Ltd., Shanghai, China). The charge–discharge tests were performed on a battery testing system (NEWARE CT-4008, Shenzhen, China) at 25 °C. The cyclic voltammetry (CV) test was performed through an electrochemical workstation (Chenhua CHI760E, Shanghai, China).

## 3. Results and Discussion

**Figure 2:**
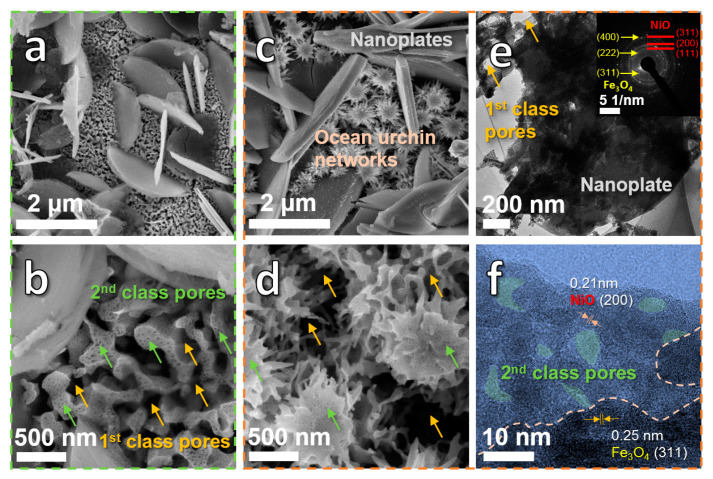
(**a**,**b**) SEM images of Ni_5_Fe_5_Al_90_ ribbons after dealloying in 2 M NaOH solution for 5 h; SEM (**c**,**d**) and TEM (**e**,**f**) images of Ni_5_Fe_5_Al_90_ ribbons after dealloying in 2 M NaOH solution for 24 h.

Figure 2a–d show the SEM images of the Ni_5_Fe_5_Al_90_ sample after de-alloying for 5 h (DA-5) and 24 h (DA-24). The DA-5 sample is composed of a dual-network structure (Figure 2a), which contains the nanoplate (iron oxide) network and the nano-ligament network. The enlarged image (Figure 2b) reveals that the ligamental network possesses a bimodal nanoporous structure, containing the first class pores (70–250 nm) marked by yellow arrows and the second class pores (10–30 nm) marked by green arrows. It is interesting to find that when the dealloying time is increased from 5 h to 24 h, the bimodal nanoporous ligamental structure transforms into urchin networks (Figure 2c). In this situation, a lot of urchins are generated from aggregation of local ligaments. Specially, the urchins are still connected by surrounding tentacles (ligamental networks), and the urchin networks also present a bimodal nanoporous structure (first class pores: 50–150 nm, second class pores: 4–20 nm) as marked by yellow and green arrows (Figure 2d). The ample pores are conducive to the transmission of ions and electrons. Moreover, the length and width of nanoplates increase slightly due to coarsening in the growth process. The TEM images (Figure 2e,f) uncover representative morphologies of nanoplates and bimodal nanoporous networks in the product. Both the inserted diffraction spots (Figure 2e) and lattice fringes (Figure 2f) demonstrate the existence of Fe_3_O_4_ and NiO. In addition, the two kinds of oxides form heterostructures in local areas with clear boundaries, which is beneficial to play a synergistic role in lithiation/de-lithiation process.

**Figure 3:**
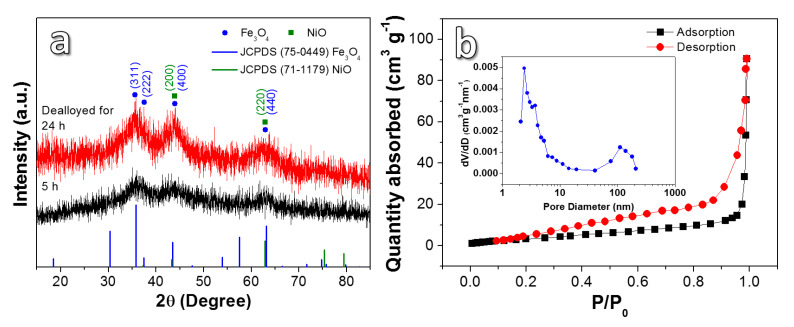
(**a**) XRD patterns of Ni_5_Fe_5_Al_90_ ribbons dealloyed in 2 M NaOH solution for different times; (**b**) N_2_ adsorption/desorption isotherm characteristics and pore size distribution of the sample dealloyed for 24 h.

The phase compositions of the as-synthesized samples were distinguished by XRD (Figure 3a). The two samples show similar peak shapes but differ in peak intensities. Although background “noise” is present in XRD, the peaks with strong intensities can be conservatively associated with the oxides by comparing Joint Committee Powder Diffraction Standards (JCPDS) cards. The diffraction peaks at 35.8°, 44.2°, and 62.9° correspond to (311), (222), (400), and (440) crystal planes of Fe_3_O_4_ (JCPDS No. 75-0449). While the diffraction peaks at 43.3° and 62.9° relate to the (200) and (220) crystal planes of NiO (JCPDS no. 71-1179). The above results further indicate the existence of a Fe_3_O_4_/NiO composite. Figure 3b shows the adsorption/desorption isotherm and pore size distribution of the DA-24. It presents a type-IV isotherm containing a type-H3 hysteresis loop [23] with corresponding specific surface area of 65.65 m^2^ g^−1^. The insets reveal two peak values of the sample in pore diameter. One is less than 10 nm and another is in the range of 70–200 nm, which demonstrates a bimodal nanoporous structure, according with the structural feature shown in SEM and TEM images. The existence of a large number of mesopores may buffer the volume expansion of the electrode during the charging/discharging process, which is beneficial to the maintenance of cyclic stability.

**Figure 4:**
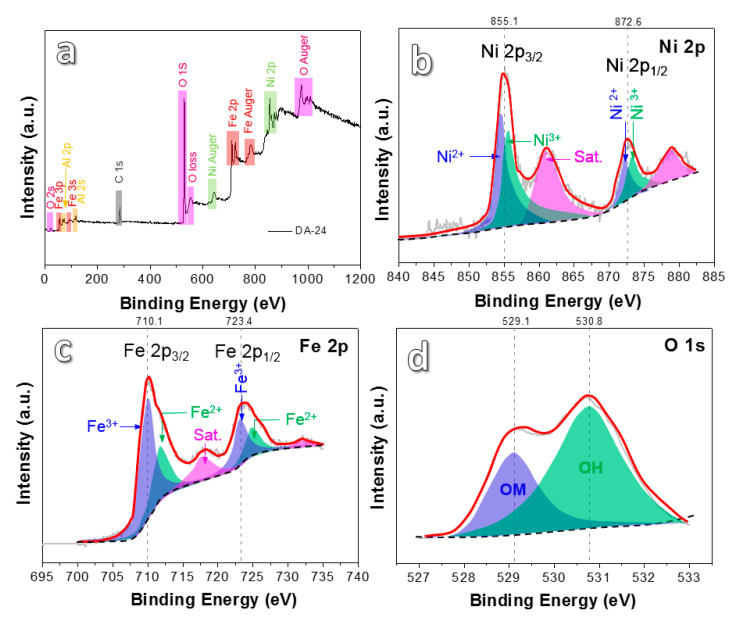
XPS spectra of the Ni_5_Fe_5_Al_90_ ribbon dealloyed for 24 h: (**a**) survey spectrum; (**b**) Ni 2p; (**c**) Fe 2p; and (**d**) O 1s.

The valence states of superficial elements in the DA-24 sample were verified by XPS analysis (Figure 4). The XPS full survey spectrum (Figure 4a) clearly reveals the presence of Ni, Fe, O elements, and undealloyed residual Al. Figure 4b presents two characteristic peaks at 855.1 eV and 872.6 eV, which correspond to Ni 2p_3/2_ and Ni 2p_1/2_ [16,26], respectively. Each characteristic peak can be deconvoluted into two peaks related to the Ni^2+^ and the Ni^3+^. Two shakeup satellite peaks at 861.6 eV and 879.8 eV can also be found. These satellites involve core ionization and simultaneous excitation of a valence electron into an unoccupied orbital, typically described as shake excitations [27]. The Fe 2p spectrum in Figure 4c shows two characteristic peaks at 710.3 eV and 723.4 eV, which are ascribed to Fe 2p_3/2_ and Fe 2p_1/2_ [26], respectively. Every characteristic peak is a superposition of two peaks including the Fe^3+^ peak and the Fe^2+^ peak. The XPS spectrum of O 1s (Figure 4d) can be deconvoluted into two peaks. The peak at 529.1 eV is attributed to OM (O–Ni and O–Fe) bonds [3], and another peak at 530.8 eV can be attributed to the OH bond, which may come from residual sodium hydroxide etchant and absorbed H_2_O from the air [28]. XPS results further confirm the existence of iron and nickel oxide species in different oxidation states.

**Figure 5:**
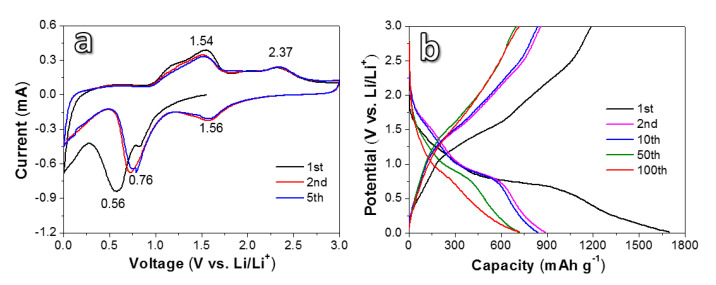
Cyclic voltammetry (CV) (**a**) and galvanostatic charge/discharge (**b**) curves of the products dealloyed for 24 h.

Figure 5a reveals the CV curves of the DA-24 sample testing at a scan rate of 0.1 mV s^−1^ in a voltage range of 0.01–3 V. In the initial reduction process, the sample shows two reduction peaks near 0.8 V and 0.6 V. The peak at 0.8 V corresponds to the reduction of NiO to Ni [29] and the peak at 0.6 V is related to the reduction of Fe^3+^ or Fe^2+^ to Fe^0^ [30]. The two reduction peaks move to about 1.56 V and 0.76 V in the later cycles. The difference in the peak position and peak intensity between the first cycle and the following cycles can be attributed to the formation of a solid electrolyte interface (SEI) layer and the structural modification of Li^+^ drive during the lithiation process [26]. In the first anodic scan, the broad peak in the range of 1.1–1.7 V can be ascribed to the step-by-step oxidation of Fe^0^ to Li_x_Fe_3_O_4_ and Fe^3+^, respectively [30]. The peak at 2.3 V corresponds to the oxidation of Ni to NiO [29]. The repeatability of the CV curves between the second cycle and the fifth cycle is very good, which reflects its good electrochemical stability and reversibility. Figure 5b shows galvanostatic charge/discharge curves of the DA-24 sample cycling at 100 mA g^−1^. The discharge curve presents two platforms of 1.2–1.7 V and 0.4–0.9 V. While in the charging process, two platforms of 1.0–1.6 V and 1.7–2.5 V can be found. These positions of platforms are consistent with CV results. With the increase in cycle number, the profile gradually moves to the left, indicating a slight decrease in capacity after several cycles. The charge/discharge profiles of DA-24 for the 50th and 100th laps are very close, revealing a good cycling stability of the electrode at the later stage.

**Figure 6:**
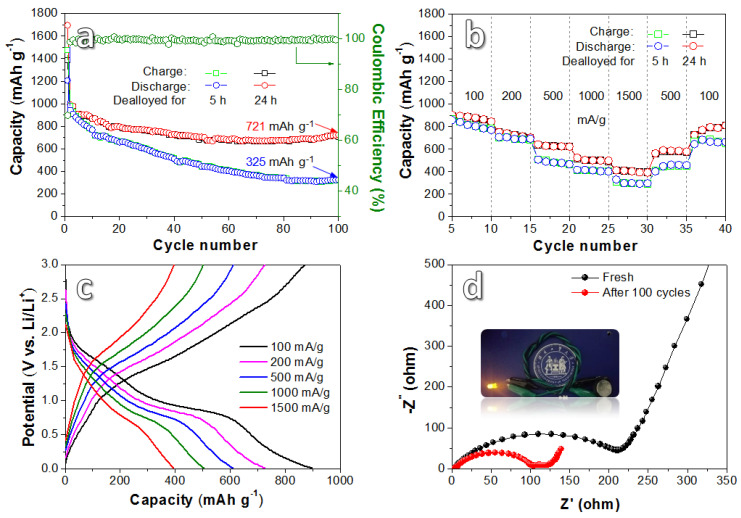
(**a**) Cycling performance of the electrode materials testing at a current density of 100 mA g^−1^ for 100 cycles; (**b**) rate performances of the products tested at different current densities; (**c**) galvanostatic charge/discharge curves of the products dealloyed for 24 h at different current densities; (**d**) electrochemical impedance spectroscopy (EIS) interceptions of the products dealloyed for 24 h.

Figure 6a shows the cycle performance of the DA-5 and the DA-24 sample cycling for 100 cycles at a current density of 100 mAg^−1^. The discharge capacity of the two samples in the first cycle is 1203 mAh g^−1^ and 1697 mAh g^−1^, respectively. The initial coulombic efficiency (CE) of the DA-24 is about 82%, which is related to the creation of a SEI film. From the fifth to the one hundredth cycle, the average CE is about 99.56%, demonstrating a relatively good electrochemical reversibility. The reversible capacity of DA-24 gradually decreases during the first 50 cycles and remains relatively stable for the following 50 cycles. While for the DA-5 sample, the reversible capacity keeps declining. After 100 cycles, DA-24 maintains a reversible capacity of 721 mAh g^−1^, while DA-5 maintains a reversible capacity of 325 mAh g^−1^, showing a better cycling stability of DA-24 than that of DA-5. It should be mentioned that compared to traditional graphite anodes (90 wt.% + active material), the transition metal oxide-typed anodes inevitably contain a lower proportion (70 wt.% in this study) of active material in the overall electrode composition, which affects the real achievable capacity of the whole electrode to a certain degree.

Figure 6b shows rate performances of the samples. For the DA-24 sample, when the current densities are 100, 200, 500, 1000, and 1500 mA g^−1^, the reversible capacities are 879, 728, 594, 493 and 386 mAh g^−1^, respectively. When the current density lowered to 100 mA g^−1^, the reversible capacity of 765 mAh g^−1^ can be maintained. In contrast, the DA-5 achieves lower capacities than DA-24 at every current density. Figure 6c shows the galvanostatic charge/discharge curves of the DA-24 anode at different current densities. It shows that with the increase in current density, the curve gradually shifts to lower capacities while the shape is basically unchanged. Figure 6d shows the electrochemical impedance spectroscopy (EIS) of the fresh DA-24 electrode and the electrode cycles for 100 cycles at 100 mA g^−1^. The EIS consists of two parts including the high and medium frequency region (semicircle) caused by charge transfer resistance and the low-frequency region (slash) because of ion diffusion. Obviously, the electrode presents lower resistance and better conductivity after cycling, which may possibly be due to structural rearrangement of surface elements and the formation of the stable SEI layer [30] during cycling process. As shown in the insert of Figure 6d, in order to evaluate the possibilities of the as-prepared Fe_3_O_4_/NiO electrode in practical application, we put a battery in series with an LED bulb (0.1 W, 3 V). The brightness of the LED bulb is high and hardly declines after working for 0.5 h.

Table 1 [8,29,31,32,33,34] presents the Li storage performance of the recently studied electrode materials. It can be seen that the Li storage performance of the as-prepared Fe_3_O_4_/NiO anode is higher than most of the reported Fe_3_O_4_- or NiO-based composites. The good Li storage performance is put down to the following points. Firstly, the Fe_3_O_4_/NiO electrode possessing a relatively high specific surface area can offer a large contact area and close interaction between the active material and the electrolyte. Secondly, the three-dimensional porous urchin network with interconnected ligaments can enhance ionic mobility and permeability. Moreover, the ample pores with bimodal structure can effectively alleviate volume expansion of active materials. In summary, Fe_3_O_4_/NiO electrode fabricated by facile process possesses excellent potential as an anode for LIBs application. In addition, this paper also offers a new idea for the design and preparation of low-cost and high-performance electrode materials with novel porous structures by dealloying method and may promote the development of the dealloying method.

## 4. Conclusions

By carefully designing the components of precursor alloys and controlling corrosion conditions, the bimodal nanoporous urchin network was successfully introduced into the dual network Fe_3_O_4_/NiO composite. The as-obtained dual network composite, containing a nanoplate array network and a bimodal nanoporous urchin network, presents good electrochemical performance as anode material for lithium ion batteries, delivering a reversible capacity of 721 mAh g^−1^ after 100 cycles at a current density of 100 mA g^−1^. Such a structural design strategy can be extended for the design and preparation of potential electrode materials with novel porous structures and may promote the in-depth development of the dealloying technology in broad areas.

## Figures and Tables

**Table 1:** The comparison of Li-ion storage performance among different works.

Materials	Current Density (mA g^−1^)	Cycle Number	Reversible Capacity (mAh g^−1^)	Reference
Flower-like NiO/RGO nanocomposites	100	100	702.3	[31]
Graphene nanosheets loaded Fe_3_O_4_ nanoparticles	100	80	600	[8]
Carbon-coated α-Fe_2_O_3_@Fe_3_O_4_	100	50	675.6	[32]
NiFe_2_O_4_/NiO@Fe_2_O_3_ core-shelled nanocubes	100	50	625.27	[33]
SnO_2_@C@Fe_3_O_4_ hollow nanospheres	100	100	540.5	[34]
Cu doped flake-NiO	100	50	655.3	[29]
Dual-network porous Fe_3_O_4_/NiO	100	100	721	This work
